# Evaluation and Application of the MIRA–qPCR Method for Rapid Detection of Norovirus Genogroup II in Shellfish

**DOI:** 10.3390/microorganisms13040712

**Published:** 2025-03-21

**Authors:** Yanting Zhu, Mengyuan Song, Yingjie Pan, Yong Zhao, Haiquan Liu

**Affiliations:** 1 College of Food Science and Technology, Shanghai Ocean University, Shanghai 201306, China; 2 Shanghai Engineering Research Center of Aquatic-Product Processing & Preservation, Shanghai 201306, China; 3 Laboratory of Quality & Safety Risk Assessment for Aquatic Products on Storage and Preservation (Shanghai), Ministry of Agriculture and Rural Affairs, Shanghai 201306, China; 4 Engineering Research Center of Food Thermal-Processing Technology, Shanghai Ocean University, Shanghai 201306, China

**Keywords:** MIRA–qPCR, rapid amplification, norovirus GII, shellfish, food safety

## Abstract

Globally, norovirus has become the primary cause of outbreaks of acute gastroenteritis, and an increasing number of norovirus GII infections have been associated with shellfish. This highlights the urgent need to establish sensitive and rapid detection platforms for timely screening of contaminated shellfish to reduce the risk of virus transmission. To address this challenge, we developed a novel detection method combining multienzyme isothermal rapid amplification (MIRA) with qPCR, referred to as MIRA–qPCR, specifically targeting norovirus GII. It exhibited robust specificity, demonstrating no cross-reactivity with *sapovirus*, *rotavirus*, hepatitis A virus, *Escherichia coli*, *Listeria monocytogenes*, or *Vibrio parahaemolyticus*, and exhibited high sensitivity, detecting as low as 1.62 copies/μL for recombinant plasmid standards. Furthermore, MIRA–qPCR showed good linearity in the 1.62 × 10^1^ to 1.62 × 10^7^ copies/μL range, with an R^2^ > 0.90. MIRA–qPCR and qPCR assays were performed on 125 fresh shellfish samples; there was good consistency in the detection results, and the Kappa value was 0.90 (*p* < 0.001). The sensitivity and specificity of the MIRA–qPCR detection were 100.00% and 97.25%, respectively. The MIRA–qPCR technique provides a viable alternative for the rapid screening of norovirus GII-contaminated shellfish to guarantee food safety.

## 1. Introduction

Globally, the societal burden of norovirus infections is significant, with estimated yearly costs exceeding USD 60 billion [1,2]. Based on the latest symptomatic cases, outbreak incidents, and sporadic community cases caused by norovirus infections in the United States, Bartsch et al. [2] evaluated that the resulting costs, including direct medical expenses and productivity losses, amount to as high as USD 10.6 billion. Currently, there is no available vaccine or antiviral treatment program for norovirus infections. Given the highly infectious nature of norovirus and the significant potential public health threat posed by food contamination with trace amounts of viruses, the sensitivity of virus detection methods is critical.

Norovirus, a single-stranded, positive-sense, non-enveloped RNA virus, belongs to the genus Norovirus in the family *Caliciviridae*. Its genome is approximately 7.5–7.7 kb and contains three open reading frames (ORF1, ORF2, and ORF3) [3]. Norovirus is rapidly evolving and genetically diverse, and studies have shown that it can be phylogenetically classified into 10 genogroups (GI-GX) and more than 49 genotypes, based on the diversity of amino acids encoded by the intact VP1 and the nucleotide diversity of the RNA-dependent RNA polymerase (RdRp) region of ORF1 [4]. Norovirus GI, GII, GIV, GVIII, and GIX can infect humans [5]. Human noroviruses (HNV) are responsible for widespread outbreaks of acute gastroenteritis and represent one of the most common causes of foodborne illness. Most human cases are associated with norovirus genogroup II (GII) [6], with genotype GII.4 being the most prevalent strain. Parikh et al. [7] analyzed the seasonal patterns and genotype distribution among sporadic norovirus gastroenteritis cases and reported norovirus outbreaks in Middle Tennessee between 2012 and 2016. Among the 755 collected cases of pediatric sporadic norovirus infection, norovirus GII accounted for the majority of cases (83.3% to 90.1%), and GII.4 was the dominant genotype (39.0% to 52.8%). Gao et al. [8] investigated the genetic characteristics of noroviruses in Beijing, where a total of 762 outbreaks were reported between September 2014 and August 2017, with GII.P16-GII.2 and GII.P17-GII.17 being the most common genotypes. It can be seen that over the past 10 years, the genogroup II noroviruses have been predominantly prevalent around the world.

Norovirus is highly contagious. Its principal modes of transmission include the ingestion of contaminated water or food and contact with infected individuals [9]. Research has demonstrated that norovirus can withstand prolonged periods on a range of food surfaces and in water, and that its transmission is facilitated by the contamination of object surfaces, foodstuffs, and water present in aerosols [10]. Norovirus outbreaks usually peak in winter and early spring (October to March). Among these transmission routes, the main route leading to norovirus infection is often through food transmission. Campos et al. [11] found that the seasonal outbreaks of norovirus in oyster populations were related to water temperature and possibly, oyster metabolism. Shellfish are often one of the types of foods at high risk of norovirus contamination because of their ability to enrich viruses from contaminated water bodies and because of people’s dietary habits of eating shellfish raw. Shellfish, such as oysters, are good hosts for norovirus. Many norovirus outbreaks have been associated with oyster consumption [12,13]. Oysters act as filter feeders and are capable of enriching virus particles in their digestive tract from contaminated water. Once raw, contaminated, or undercooked oysters are consumed, the intact virus particles can be easily transmitted to consumers, resulting in mass foodborne illness [14,15,16,17].

Molecular biology techniques centered around the detection of viral nucleic acids have become routine laboratory procedures for norovirus identification [18,19,20]. These methods encompass RT-PCR and nucleic acid probe hybridization. However, they involve temperature cycling, are time-consuming, require complex protocols, are technically demanding for operators, and are susceptible to external environmental influences [21,22,23]. In recent years, thermostatic amplification techniques have developed rapidly due to their relative simplicity and greater speed in obtaining results. Multienzyme isothermal rapid amplification (MIRA), a thermostatic nucleic acid amplification technique, produces results in just 5–20 min [22,24,25,26,27]. MIRA has been utilized for the detection of SARS-CoV-2 [28], hepatitis A virus (HAV) [29], and bovine coronavirus [30].

There are few previous reports of MIRA application in norovirus, so in this study, a novel, ultra-fast assay method was established by combining MIRA with the qTOWER^3^G qPCR instrument. MIRA–qPCR avoids the drawbacks of the above methods through a one-step process from amplification to readout, and the amplification process takes only 20 min at a constant temperature, which is a great advantage for rapid detection. The accuracy of the MIRA–qPCR method was assessed using specificity, sensitivity, and repeatability experiments. After the basic accuracy of the MIRA–qPCR method was validated through specificity, sensitivity, and repeatability experiments, it was essential to further evaluate its practical diagnostic capabilities in real samples. The diagnostic performance of MIRA–qPCR was assessed by applying it to commercially available raw shellfish and comparing it with the performance of qPCR. This method provides technical support for the safety risk assessment of shellfish and other aquatic products that may be contaminated with norovirus.

## 2. Materials and Methods

### 2.1. Norovirus GII Deoxyribonucleic Acid Standard Preparation

Since there is no suitable in vitro cell culture system that can culture human norovirus, the complete gene sequence of norovirus GII was downloaded from the NCBI database, and a sequence comparison analysis was performed using DNAMAN software 9.0 to obtain the relatively conservative gene sequence regions provided in Figure S1. A 480 bp DNA fragment from norovirus GII was cloned into the plasmid pUC57(+), from Sangon Biotech (Shanghai) Co., Ltd., Shanghai, China, and glycerol-preserved bacteria. The synthetic sequence is shown in Table S1.

After extracting the plasmid using the TIANGEN plasmid miniprep kit (TIANGEN, Beijing, China), as per the manufacturer’s instructions, the concentration and purity of recombinant plasmid DNA were measured with a NanoDrop™ One ultra-micro UV spectrophotometer (Thermo Fisher Scientific, Waltham, MA, USA), according to the following copy number calculation formula: DNA copy number (copies/μL) = [6.02 × 10^23^ × plasmid concentration (ng/μL) × 10^−9^]/[plasmid bases (nt) × 660] [31].

### 2.2. Design of Primers and Probes

Primers were designed following the instructions of the DNA Fluorescent Kit (Amp-future, Weifang, China) The highly conserved sequence of norovirus GII (GenBank number: X86557) was selected. Based on primer screening, a 46–50 nt probe was located between the upstream and downstream primers. The 5′ end of the probe was modified with 6-FAM; there was a dSpacer (tetrahydrofuran, THF, Sangon Biotech (Shanghai) Co., Ltd.) with fluorescent and quenching groups on either side of it. The probe was modified with a C3-Spacer approximately 15 nt from the 3′ end. The lengths of the target amplification products ranged from 150 to 300 bp. All primers and probes were designed using Primer Premier 5 software, except for the qPCR primers and probes, which were derived from Annex D (informative) of ISO 15216-1:2019 [32]. Primer specificity was verified using NCBI’s Primer-BLAST (https://blast.ncbi.nlm.nih.gov/Blast.cgi, accessed on 11 March 2024).

### 2.3. Principle and Workflow of MIRA–qPCR Assay

**Figure 1:**
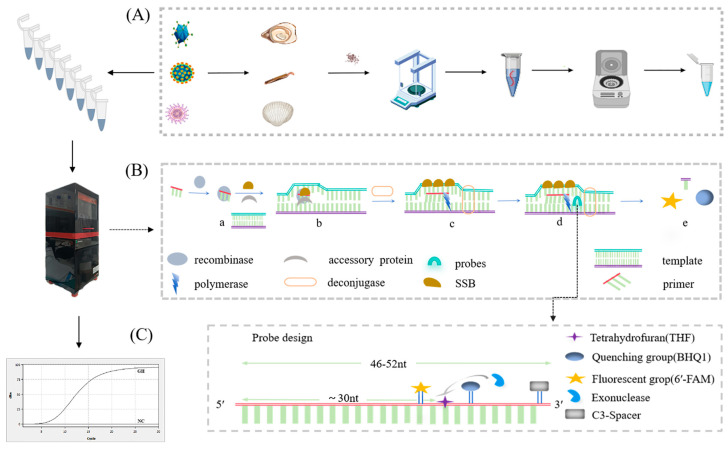
Schematic diagram of the process of rapid detection of norovirus GII using MIRA–qPCR technology. The detection process is divided into three steps: (**A**) RNA extraction and reverse transcription, created with MedPeer.com. (**B**) Gene amplification and fluorescence signal acquisition by MIRA–qPCR, i.e., a. the Rec/SSDNA complex is formed; b. invasion of the template and forming of a D-loop region; c. DNA strand extension; d. probe hybridization; e. release of fluorescent groups. (**C**) Curve amplification obtained and results read.

Using MIRA–qPCR, we were able to amplify the samples within 20 min at a constant temperature of 39–42 °C. The principle behind the MIRA–qPCR assay is shown in Figure 1B. Recombinant enzymes and primers form the protein/single-stranded nucleotide complex Rec/ssDNA, which invades the double-stranded DNA template with the help of auxiliary proteins and the single-stranded binding protein, SSB. A D-loop region was formed at the invasion site, and the double-stranded DNA was scanned; after finding the target region that was complementary to the primer, the recombinant Rec/ssDNA complex disintegrated, and the polymerase bound to the 3′ end of the primer to start strand extension. Finally, the THF site was recognized by the nucleic acid exonuclease and hydrolyzed, the fluorescent groups were released, and the fluorescence monitoring device of the qPCR instrument monitored the amplification process of the target fragment in real time. The level of the negative fluorescence amplification curve indicated the absence of the target gene, and the appearance of the fluorescence amplification curve indicated the presence of norovirus GII. If the reported Ct value is greater than 35 or is otherwise indeterminate, it is to be inferred that the result is negative. The entire process, from sample preparation (Figure 1A) to target gene amplification (Figure 1B) and interpretation of the results (Figure 1C), takes approximately one hour.

### 2.4. MIRA–qPCR on Analytik Jena’s qTOWER^3^G System

MIRA–qPCR was performed using the qTOWER^3^G Touch instrument (Analytik Jena AG, Jena, Thüringen, Germany) at a constant temperature, and the FAM channel was set to read fluorescence every 30 s. Nucleic acid amplification was performed using the MIRA fluorescence kit (Amp-future, Weifang, China). According to the manufacturer’s instructions, the reaction system contained 29.4 µL of A buffer, 2 µL of 10 µM each of forward and reverse primers, 0.6 µL (10 µM) of probe, 9.5 µL ddH_2_O, and 4 µL of template preparation premix, to which the 2.5 µL of B buffer (starter reagent) was added, and the reaction was initiated. The reaction solution was centrifuged to the bottom of the tube by shaking it up and down 7–8 times and centrifuging it immediately, followed by placing the reaction tube in a thermostat apparatus. Different reaction temperatures (38, 39, 40, 41, and 42 °C) were explored to determine the optimal temperature for the MIRA–qPCR reaction. Fluorescence amplification curves were analyzed using qPCRsoft 4.1, the software that comes with the Jena qTOWER^3^G PCR fluorescence quantification instrument (the qTOWER^3^G Touch instrument) (Analytik Jena AG, Jena, Thüringen, Germany).

Conventional PCR was performed on a Biometra TONE PCR instrument (Analytik Jena AG, Jena, Thüringen, Germany) using the following protocol: 5 min at 94 °C, followed by 35 cycles of 30 s at 94 °C, 30 s at 57.1 °C, 30 s at 72 °C, and then a final extension at 72 °C for 10 min to complete the amplification. The 25 µL of the reaction mixture was prepared, consisting of 12.5 µL of 2 × Taq Master Mix with blue dye (Sangon Biotech (Shanghai) Co., Ltd., Shanghai, China), 1 µL of 10 µM forward primer, 1 µL of 10 µM reverse primer, 2 µL of template, and the remaining volume made up with ddH_2_O. PCR amplification products (3 µL per sample) were electrophoresed on a 2% (*w*/*v*) agarose gel at 120 V for 50 min and then visualized using an Invitrogen iBright gel imager (Thermo Fisher Scientific, Waltham, MA, USA). The amplification products of basic MIRA could also be used for the agarose gel electrophoresis analysis described above.

### 2.5. Accuracy Assessment of MIRA–qPCR Method

For MIRA–qPCR, the DNA templates of the genomic fragments of *sapovirus* (SV), *rotavirus* (RV), and hepatitis A virus (HAV) were synthesized by Sangon Biotech and diluted with ddH_2_O gradient to the concentration of 10^5^ copies/μL. The synthetic sequence is shown in Table S2. The genomic DNA of the laboratory-stored *Escherichia coli* (*E. coli*), *Listeria monocytogenes* (LM), and *Vibrio parahaemolyticus* (VP) was extracted from their respective bacterial suspensions of 10^5^ CFU/mL using the Bacterial Genomic DNA Extraction Kit (TianGen, Beijing, China), according to the manufacturer’s instructions. The genomic DNA was used as the template, with ddH_2_O as a negative control.

In the NCBI-BLAST (https://blast.ncbi.nlm.nih.gov/Blast.cgi, accessed on 13 May 2024) simulation, the potential cross-reactivity of the optimal primer set was evaluated using the input template with accession number X86557.1. This optimal primer set was matched against the template and the organisms within the Core Nucleotide, which included *sapovirus*, Lordsdale virus, norovirus GI, norovirus GIV, human rotavirus A, and Hepatovirus A virus.

The sensitivity analysis of the MIRA–qPCR assays was performed using 10-fold serial dilutions of recombinant plasmid standards ranging from 1.62 × 10^7^ to 1.62 × 10^−1^ copies/μL as a template and ddH_2_O as a negative control. The qPCR assay was also conducted using PerfectStart^®^ II Probe qPCR SuperMix (TRAN, Beijing, China) for comparison. The rapid reaction procedure consisted of 30 s at 95 °C, followed by a total of 40 cycles at 95 °C for 5 s and 60 °C for 30 s, during which the fluorescent signal was collected, according to the manufacturer’s instructions. All amplification reactions were repeated three times. Conventional PCR was used as a control.

Three gradient-diluted concentrations of the extracted recombinant plasmid standards were selected for three replicate experiments. Each test concentration was repeated three times independently, and the coefficient of variation (CV) value was calculated based on the cycle threshold (Ct value) of the test results to assess the reproducibility [33].

### 2.6. Detection of Norovirus in Shellfish Food via MIRA–qPCR

All of the fresh shellfish were purchased from the seafood market in Shanghai at the same time. The 125 food samples comprised 25 clams, 25 razor clams, 25 black mussels, 30 oysters, and 20 scallops. They were transported in ice packs and placed in ice baths in a sterile sampling bag immediately after retrieval. The digestive gland tissue of each weighed shellfish was immediately placed in the lysis buffer RLT Plus. RNA was extracted from the samples via the Modified Tissue/Cell RNA Rapid Extraction Kit (SparkJade, Shandong, China), and the remaining samples were stored in a −80 °C refrigerator for one week. The nucleic acids were quantified for concentration and purity using a NanoDrop™ One ultra-micro UV spectrophotometer, followed by reverse transcription into cDNA via TransScript One-step gDNA Removal cDNA Synthesis SuperMix (TRAN, Beijing, China), according to recommended procedures. MIRA–qPCR and qPCR assays were performed on 125 samples. To assess the MIRA–qPCR method, the Kappa coefficient was analyzed using IBM SPSS Statistics 26 software.

## 3. Results

### 3.1. Primer Sets Screened and Systems Optimization

**Figure 2:**
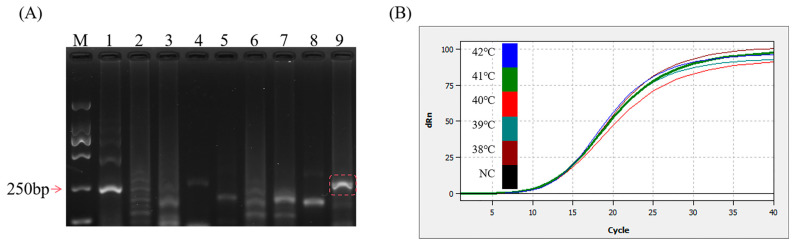
Optimization of reaction conditions. (**A**) 2% gel plot for basic MIRA screening primers. M: D2000 marker—lane 1: Nf1/NR1; lane 2: Nf1/NR2; lane 3: Nf1/NR3; lane 4: Nf2/NR1; lane 5: Nf2/NR2; lane 6: Nf2/NR3; lane 7: Nf3/NR2; lane 8: Nf3/NR3; lane 9: Nf3/NR1. (**B**) MIRA–qPCR temperature optimization: 10^5^ copies/μL were selected and amplified at 38, 39, 40, 41, and 42 °C for 20 min. NC: negative control.

All the sequences of the primers and probes used in this study are displayed in Table 1. Using 1.62 × 10^4^ copies of the DNA standard as the template, the basic MIRA amplification of nine sets of primer pairs was performed at 37 °C for 30 min using the incubation procedure of the Biometra TONE PCR instrument (Analytik Jena AG, Jena, Thüringen, Germany), and a set of primers with the best performance was selected. As shown in Figure 2A, lane 9 was free of spurious bands and displayed a higher brightness when compared to that of other lanes. The amplification length of 267 bp, which is above 250 bp, aligns with the theoretical value. Therefore, the Nf3/NR1 primer pair was identified for subsequent experiments. The MIRA–qPCR amplification curves within the 38–42 °C range were close, as shown in Figure 2B, and 41 °C was selected, based on the Ct values.

### 3.2. Accuracy Analysis of MIRA–qPCR Method

**Figure 3:**
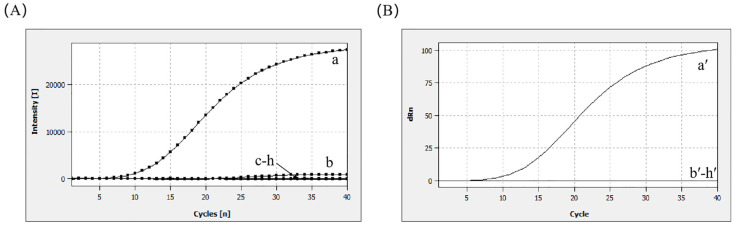
Specificity analysis. (**A**) a: 10^5^ copies/μL (norovirus GII); b: 10^5^ copies/μL (SV); c: 10^5^ copies/μL (HAV); d: 10^5^ copies/μL (RV); e: 10^5^ CFU/mL (*E. coli*); f: 10^5^ CFU/mL (LM); g: 10^5^ CFU/mL (VP); h: negative control. (**B**) The Ct plot was calculated to correspond to the amplification plot shown in (**A**).

The DNA genomes of common enteric diarrhea viruses and foodborne pathogens were tested using the MIRA–qPCR assay. The results of the MIRA–qPCR assay revealed that the amplification curve and the corresponding Ct value were observed only for norovirus. There was no Ct value, despite the slight upward detection of the amplification curve of SV in Figure 3. No amplification signal was detected for RV, HAV, *E. coli*, LM, VP, and ddH_2_O. Using MIRA–qPCR, only norovirus GII showed positivity, and there was no cross-reactivity (Figure 3B). The results showed that MIRA–qPCR exhibited high specificity for the detection of norovirus.

Through NCBI-BLAST analysis, Nf3/NR1 was found to match perfectly with the target sequence, exhibiting good specificity. Only the nonstructural polyprotein of norovirus GI isolate 89 GI.3 showed potential for non-specific amplification, as there were 4–5 mismatched bases between it and the primers. Most of these mismatched bases were thymine (T) bases and required higher energy for base-pairing, as shown in Figure S2. It is assumed that the probability of false positives is low.

**Figure 4:**
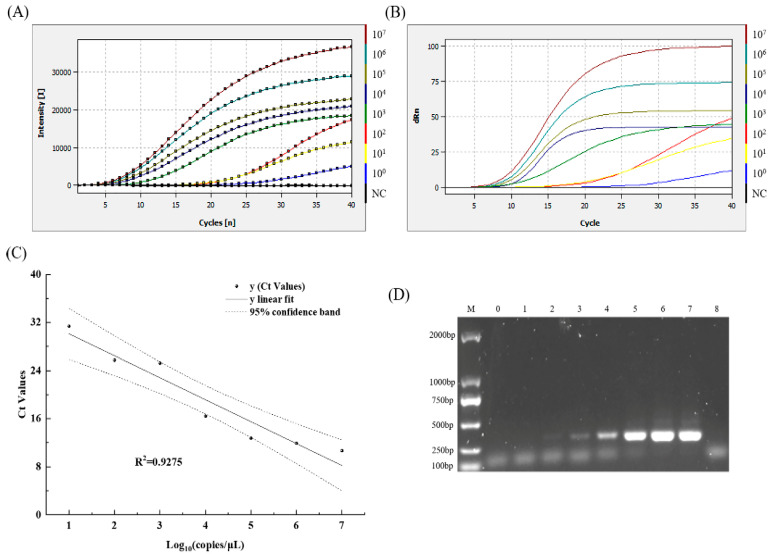
The LOD-relevant results of norovirus GII using MIRA–qPCR and conventional PCR. (**A**) The amplification curves show the LOD of MIRA–qPCR. NC: negative control. (**B**) The Ct plot was calculated to correspond to the amplification plot shown in (**A**). (**C**) The linear regression standard curve between the Ct values and the logarithm concentrations of norovirus GII, as well as the 95% confidence band, were obtained using the software Origin 2021. (**D**) The LOD of conventional PCR. M: D2000 Marker, lanes 0–7; the corresponding concentrations of norovirus GII were 1.62 × 10^0^~10^7^ copies/μL; lane 8: negative control.

The sensitivity of the MIRA–qPCR assay was tested with 10-fold serial dilutions of plasmid DNA standard with concentrations ranging from 1.62 × 10^7^ to 1.62 × 10^0^ copies/μL. As the concentration of plasmid DNA decreased, the fluorescence intensity decreased, and the Ct value increased. MIRA–qPCR detected 1.62 × 10^0^ copies/μL, as shown in Figure 4A, and the concentration of 1.62 × 10^−1^ copies/μL was undetectable, as shown in Figure S3, in which the amplification curve overlaps with the negative control. The LOD of the MIRA–qPCR was 1.62 copies/μL, and the corresponding Ct value was 34.62 (Figure 4A,B). A good linear relationship was exhibited in the concentration range of 1.62 × 10^7^ to 1.62 × 10^1^ copies/μL. The log concentration of the norovirus GII was fitted as y=−3.653x+33.771 (R^2^ > 0.9). The amplification efficiency of MIRA–qPCR was 87.8%, according to the formula $E=10^{-1∕slope}-1$. The MIRA–qPCR values were all within the 95% confidence band, indicating that the quantitative results were accurate within the 95% detection limit (Figure 4C). The lowest quantitative detection line was 10^1^ copies/μL. The sensitivity of MIRA–qPCR was 100 times higher than that of conventional PCR under similar detection conditions (Figure 4D), while it was equivalent to that of qPCR (Figure S4).

To analyze the reproducibility of MIRA–qPCR, three independent replicates were performed, and the Ct values corresponding to the fluorescence amplification curves were statistically analyzed. Different batches of templates from the same gradient showed good reproducibility, with CV values ranging from 2.03% to 12.09% due to high sensitivity (Table 2).

### 3.3. Application of MIRA–qPCR Assays of Norovirus in Shellfish Foods

The applicability and validity of RT-MIRA–qPCR for testing norovirus in raw shellfish products were assessed. Some studies have shown that viruses in shellfish are mainly located in the digestive glands, inlets, outlets, gills, etc. Among them, the digestive glands are often used as the preferred tissues for shellfish virus extraction [34,35]. The 125 fresh shellfish samples were tested using both MIRA–qPCR and qPCR (Table 3). The sensitivity and specificity of the MIRA–qPCR assay were approximately 100.00% and 97.25%, respectively, and the Kappa value was 0.900 (*p* < 0.001). This indicates that the MIRA–qPCR and qPCR have excellent consistency.

## 4. Discussion

Norovirus is one of the main pathogens causing human non-bacterial gastroenteritis [13]. The foodborne transmission route, one of the common factors for norovirus outbreaks [36], poses a potential threat to public health. Therefore, there is an urgent need to develop efficient and rapid norovirus detection strategies to reduce or prevent the spread of norovirus. Currently, the diagnostic methods for detecting norovirus exhibit problems such as a long detection time and poor sensitivity, which greatly limit their effectiveness in practical applications.

In this study, we innovatively developed a combined technique using multienzyme isothermal rapid amplification and a qPCR instrument (MIRA–qPCR). By measuring the highly conserved region between ORF1 and ORF2, we achieved precise detection of norovirus GII. Compared to the traditional RT-PCR method [37], MIRA–qPCR significantly shortened the detection time for the target genes. The amplification detection takes only 20 min. This technique can rapidly and efficiently detect pathogens under isothermal conditions without agarose gel electrophoresis. The dependence on equipment is significantly lower than that of traditional PCR methods. This holds significant importance for testing scenarios with limited resources, such as the routine monitoring of foodborne disease pathogens in primary healthcare institutions.

When it comes to primer design, MIRA technology provides significant advantages over loop-mediated isothermal amplification (LAMP) technology. The LAMP reaction requires three pairs of primers [38,39]. This increases the complexity of the experimental design, particularly regarding loop primer design, but also raises the potential risk of non-specific amplification. The common endpoint detection methods used in LAMP, such as metal ion indicators and colloidal gold test strips [40,41], are prone to aerosol contamination when the lid of the reaction vessel is opened, which raises the chances of false-positive detection results. In contrast, MIRA technology can quickly diagnose pathogens using just two pairs of primers. In this study, the MIRA–qPCR method has successfully integrated detection and amplification. By incorporating a probe based on a pair of primers, the specificity and sensitivity of the detection have been improved.

Fukuda et al. [42] developed a single-tube RT-LAMP technique for the detection of norovirus GI and GII at 62 °C in 60–90 min, with a sensitivity of 10^2^ and 10^3^ copies per tube, respectively. Luo et al. [40] used RT-LAMP, combined with hydroxylated naphthalene blue (HNB) dye, for colorimetric detection of norovirus GII. The reaction was incubated at 65 °C for 60 min and then heated at 80 °C for 5 min in a turbidimeter. It was detected colorimetrically, with a sensitivity of 10^3^ copies/reaction. However, the amplification temperature of LAMP (60–65 °C) is relatively high compared with that of MIRA–qPCR (39–42 °C). In this study, the MIRA–qPCR method only requires 20 min for amplification, which reduces the energy consumption of the equipment, along with the labor costs, and the requirements of the equipment are lower than those of LAMP.

In this study, the MIRA–qPCR method showed good specificity in detecting norovirus GII (Figure 3). Based on the NCBI-BLAST simulation, it was found that the primer pair Nf3/NR1 has a high specificity for the target virus and a low cross-reactivity rate with norovirus GI. However, this simulation also displays certain limitations: it relies on the selected viral sequence data, and if the data are incomplete, the analysis results may be inaccurate; furthermore, the amplification conditions of the primer probes in the MIRA–qPCR were ignored. In this study, the experimental results (Table 3) showed a small number of false-positive results compared with those of qPCR in shellfish, indicating the potential cross-reactivity of this primer with some non-target viruses. Therefore, in practical applications, it is necessary to optimize the primer design or increase specific screening steps to reduce the occurrence of false-positive results.

Highly sensitive norovirus assays can provide early warning of norovirus infection. In terms of methodological evaluation, the first application of the MIRA–qPCR method was in the qTOWER^3^G Touch instrument (Analytik Jena AG, Jena, Thüringen, Germany). The MIRA–qPCR method required an amplification time one-fifth that of conventional PCR, and its sensitivity was 10^3^ times higher than that of routine PCR (Figure 4D). Moreover, the quantitative detection limit of MIRA–qPCR is consistent with that of qPCR (Figure S4). Jia et al. [43] established and evaluated the one-step RT-RPA-LFD detection of HNV GII in boiled human fecal samples, with a reaction sensitivity of 50 copies of the norovirus genome. The MIRA–qPCR method displays higher sensitivity, which may be because the recombinase of MIRA is derived from *Streptomyces coelicolor* recA (SC-recA), improving the amplification efficiency, along with RPA (T4 UvsX) [44]. The MIRA-LFD method was developed by Xu et al. to detect duck hepatitis B virus [45], with a detection limit of 45.6 copies per reaction, and the templates were extracted from serum samples. The entire detection process takes only 15 min. Although the detection time of MIRA-LFD is three-quarters that of the MIRA–qPCR method used in this study, the detection limit of the MIRA–qPCR method is 10 times higher than that of the former. The real-time RT-RPA assay for detecting norovirus GII, which was created by Han et al. [46], displayed a sensitivity of 1.66 × 10^2^ copies/μL, and its template was derived from the RNA synthetic plasmid. This suggests that the synthetic plasmid used as the template was different from the template derived from the serum samples and feces. The former is free from the interference of the sample matrix, thus resulting in more optimal detection sensitivity.

Bivalve shellfish, such as oysters, accumulate and concentrate foodborne viruses and other microorganisms through filter feeding [36,47,48]; food matrices are complex in composition and low in viral content. Only the lysis step was slightly adjusted to achieve full release of norovirus from the tissue. A total of 25 mg of digestive gland tissue from each shellfish was placed in 600 μL of lysate RLT Plus in a sterile enzyme-free 1.5 mL centrifuge tube, shaken vigorously for 2 min, and the RNA was extracted. The present study extracted the total RNA directly from the digestive gland tissues. Compared with the ISO standard method of norovirus enrichment, concentration, and RNA extraction in bivalve mollusks, this method simplifies the sample pretreatment.

Shellfish are important vectors for norovirus and HAV. The risks associated with shellfish consumption are greater if shellfish products are consumed raw or lightly cooked [49]. Although MIRA–qPCR exhibits the advantages of sensitive and rapid detection of norovirus GII compared with those of traditional PCR and gold-standard RT-qPCR, it still requires further improvement. The actual detection sensitivity of this method in shellfish and other aquatic products requires further in-depth investigation. As the samples tested are limited to bivalve mollusk shellfish, the scope of testing can subsequently be expanded to apply to fresh produce in order to serve the market and ensure food safety.

## 5. Conclusions

In conclusion, this study developed a new method of rapid and sensitive detection of norovirus GII using MIRA–qPCR technology. The MIRA reaction was completed by incubation at 41 °C for 20 min, after which the results were acquired and read according to the fluorescence amplification curve of the qTOWER^3^G Touch instrument (Analytik Jena AG, Jena, Thüringen, Germany). The MIRA–qPCR method was developed for the detection of norovirus GII nucleic acids, and it exhibits the advantages of simple operation, rapid amplification, excellent sensitivity, and good specificity. This study enriched the nucleic acid detection method for norovirus. It offers great potential for practical applications to identify and screen norovirus-contaminated shellfish and other fresh foods, ensuring food safety.

## Figures and Tables

**Table 1:** Related primer probe sequence information.

Name		Sequences (5′ → 3′)	Sites (X86557:4981–5460)
MIRA-qPCR qPCR PCR	Nf1 Nf2 Nf3 NR1 NR2 NR3 P1 * QNIF2 COG2R P2 PcrF PcrR	TGGCTCCCAGCTTTGTGAATGAAGATGGCG TGAGCACGTGGGAGGGCGATCGCAATCTGG TCTCAGATCTGAGCACGTGGGAGGGCGATC AGCGTTTCTAGGGGACACTGTGAACTCTCC TTTGTTGGCCCGCCACAGGTGCCGCAATAG GCCGCAATAGCGGCACCAACAACGGGCTCC GATGGGTCCGCAGCCAACCTCGTCCCAGAGG /i6FAMdT/CA/idSp//iBHQ1dT/AATGAGGATGTT CAGRTGGATGAGRTTCTCWGA TCGACGCCATCTTCATTCACA 5′ 6-FAM-AGCACGTGGGAGGGCGATCG-TAMRA-N3′ CAGATCTGAGCACGTGGGAG GGAGCGTTTCTAGGGGACAC	(5068–5097) (5041–5070) (5032–5061) (5268–5298) (5192–5221) (5172–5201) (5119–5168) (5012–5100) (ISO-2019) (5034–5053) (5281–5299)

* Probe markers: 32th T-base markers for 6-FAM, as/i6FAMdT/; 36th T-base markers for BHQ1, as/iBHQ1dT/; 35th base substitution, THF, as /idSp/, 3′ end markers C3-Spacer.

**Table 2:** Repeatability of the MIRA–qPCR for norovirus GII.

Concentration (Copies/μL)	Ct Values for Replicate1 (*N* = 3.51)	Ct Values for Replicate2 (*N* = 1.23)	Ct Values for Replicate3 (*N* = 1.86)	Inter-Assay Reproducibility		
				Mean of Ct Values	SD of Ct Values	Ct CV (%)
N × 10^7^	7.87	8.02	9.73	8.54	1.03	12.09
N × 10^6^	9.00	8.49	10.35	9.28	0.96	10.35
N × 10^5^	9.39	9.50	11.02	9.97	0.91	9.14
N × 10^4^	13.14	10.51	12.95	12.20	1.47	12.02
N × 10^3^	14.41	13.81	16.16	14.79	1.22	8.26
N × 10^2^	25.12	24.87	25.86	25.28	0.51	2.03
N × 10^1^	28.86	29.96	28.43	29.08	0.79	2.72

**Table 3:** Analysis of MIRA–qPCR and qPCR screening of 125 shellfish for norovirus GII.

Detection Results	Methods		Statistical Analysis	
	MIRA–qPCR	qPCR	Kappa (k)	*p*-Value of Kappa
Positive (+)	19	16	0.900	<0.001
Negative (−)	106	109		

## Data Availability

The original contributions presented in the study are included in the article/Supplementary Materials, further inquiries can be directed to the corresponding authors.

## Supplementary Materials

The following supporting information can be downloaded at: https://www.mdpi.com/article/10.3390/microorganisms13040712/s1, Figure S1. The sequence comparison analysis results. (A) The comparison of the gene sequences between the norovirus GII and GII subtypes; (B) The comparison of the gene sequences among the norovirus GII, Common GI subtypes, and Enterovirus. Figure S2. Simulation of the Specificity of the Nf3/NR1 Primer set. Figure S3 The sensitivity analysis of the MIRA-qPCR at low concentrations (A,B) 1–5: the corresponding concentration of norovirus GII is 1.62 × 10^3^~10^−1^ copies/μL, 6: negative control; (B) The Ct plot was calculated to correspond to the (A) amplification plot. Figure S4. The LOD-relevant results of norovirus GII using qPCR (A) The amplification curves showed the LOD of qPCR. (B) The Ct plot was calculated to correspond to the (A) amplification plot. (C) The linear regression standard curve between the Ct values and the logarithm concentrations of norovirus GII as well as the 95% confidence band were obtained using the software Origin 2021. Table S1. Selected relatively conservative sequences. Table S2. The synthesized fragments sequence information of the RV, SV, and HAV.
