# Supplementary material for: Evaluation and Application of the MIRA–qPCR Method for Rapid Detection of Norovirus Genogroup II in Shellfish
Source: Microorganisms. 2025 Mar 21;13(4):712. doi: 10.3390/microorganisms13040712 (PMC12029516; doi:10.3390/microorganisms13040712)
Supplement: Supplementary file 1 [file microorganisms-13-00712-s001.zip › microorganisms-3455173-supplementary.pdf]

microorganisms

Supplementary Materials:

(A)

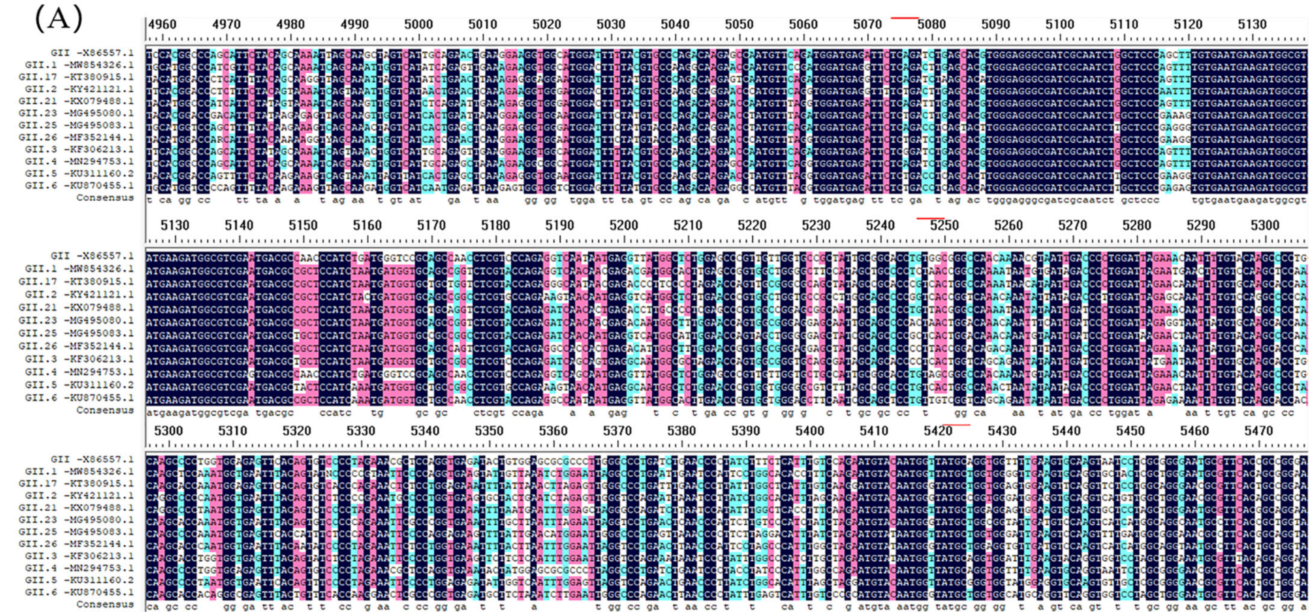

(B)

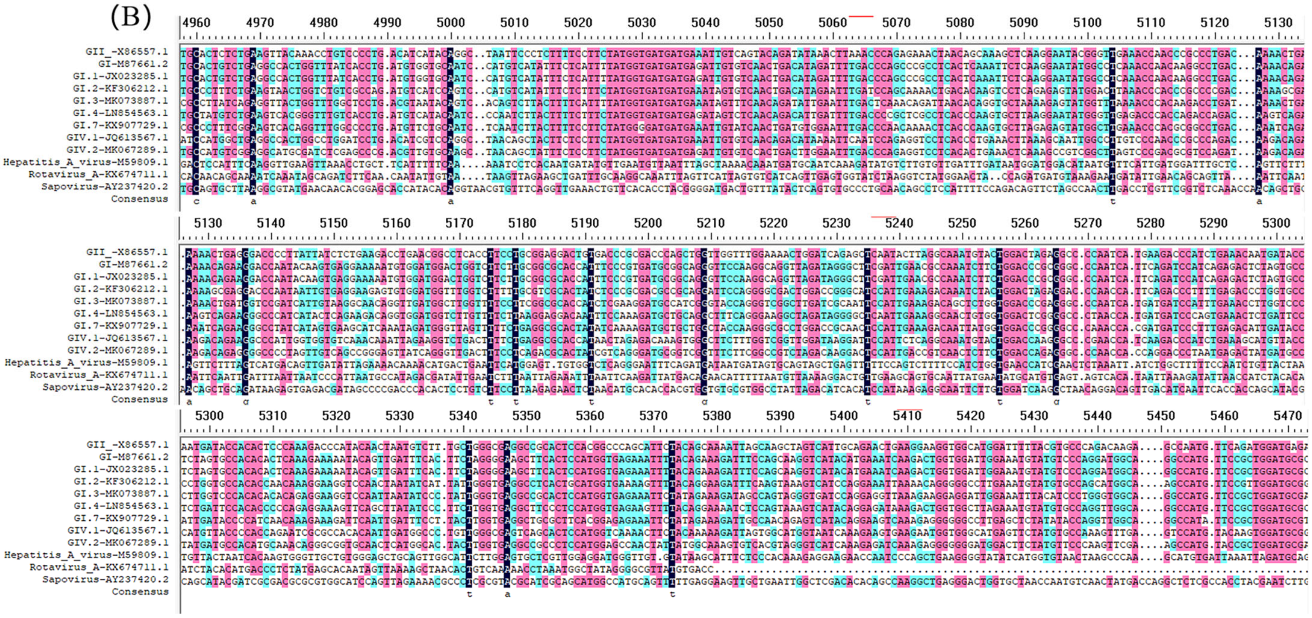

Figure S1. The sequence comparison analysis results. (A)The comparison of the gene sequences between the norovirus GII and GII subtypes; (B) The comparison of the gene sequences among the norovirus GII、Common GI subtypes, and Enterovirus.

Table S1. Selected relatively conservative sequences.

sequences	length
catggatttttacgtgcccagacaagagccaatgttcagatggatgagattctcagatctgagcacgtgggagggcgatcgcaatctg<br>gctcccagctttgtgaatgaagatggcgtcgaatgacgccaacccatctgatgggtccgcagccaacctcgtcccagaggtcaataat<br>gaggttatggctctggagcccgttgttggtgccgctattgcggcacctgtggcgggccaacaaaacgtaattgacccctggattagaa<br>acaattttgtacaagcccctggtggagagttcacagtgtcccctagaaacgctccaggtgagatactgtggagcgcgcccttgggccc<br>tgatctgaacccctatctttctcatttgtccagaatgtacaatggttatgcaggtggttttgaagtgcaagtaatcctcgcggggaatgcg<br>ttcaccgccgggaaagtcatatttgcagcagtcccac	480 (bp)

Table S2. The synthesized fragments sequence information of the RV, SV, and HAV.

name	sequences	length
Human rotavirus A (KC139781.1)	CTTAAATTTGGCCGTAAAACGATATTTTCAACTAAGAAAAATATGCATGTTATGGATGATATCGCACAT<br>GGAAGATATACTCCGGGTGTCATTCCTCCAGTGAATGTAGATAGACCAATTCCACTAGGTCGTAGAGA<br>TGTTCCTGGGCGAAGAACAAGAATTATATTCATATTGCCATATGAATACTTTATTGCGCAGCACGCTGT<br>AGTAGAAAAAATGTTATCATACGCAAAACACACTAGAGAGTACGCAGAATTCTACTCTCAGTCAAAT<br>CAATTGCTATCATATGGTGATGTTACAAGATTCTTATCTAGTAATTCTATGGTATTATACACAGATGTTTC<br>GCAATGGGATTCGTCACAACATAACACGCAACCATTTAGAAAAGGAATAATTATGGGTTTAGATATGC<br>TATCTAATATGACTAATGATCCAAAAGTAGTGCAGACGTTAAATTTATATAAACAAACACAAATTAAT<br>ATAGGTGGTACAGTACCTGACCCAGTGGGTTACACTGAAGGAACCCACAAAATAGTGTTTGAGATGG	480 （bp）
Sapovirus Mc114 (AY237422.3)	AGGGCAATGGCTCCAACTCAGAGCCAAAGCAGAGCAACAACCCAATGGTCGTTGACCCGCCTGGC<br>ACAACAGGTCCGACCACATCCCACGTTGTTGTTGCTAATCCGGAGCAACCCAATGGGGCCGCACAG<br>CGCCTGGAGTTGGCTGTTGCCACTGGTGCAATCCAATCCAATGTCCCTGAGGCAATACGCAACTGCT<br>TTGCAGTCTTTCGTACTTTTGCTTGGAACGACAGGATGCCCACGGGAACTTTTCTTGGATCTATATCGC<br>TTCATCCCAACATTAACCCGTACACTTCTCACCTCTCTGGGATGTGGGCCGGGTGGGGCGGCAGTTTT<br>GAGGTCCGGCTATCGATCTCTGGTTCTGGCGTGTTCGCTGGGCGCATCATTGCTTCTGTCA	463(bp)
Hepatitis A virus （AY334041.1）	GTTGGAGACGATTCAGGGGGTTTTTCAACAACAGTTTCTACAGAGCAGAATGTTCCTGATCCTCAGAT<br>CGGCATAACAACCATGAGGGACCTAAAAGGGAAAGCCAATAGGGGGAAGATGGATGTTTCAGGAG<br>TGCAGGCACCTGTGGGAGCTATTACAACAATTGAGGATCCAGTTTTAGCAAAGAAAGTGCCTGAGAC<br>ATTTCCTGAATTGAAGCCTGGAGAGTCCAGACATACATCAGATCATATGTCTATTTATAAATTCATGGG<br>AAGGTCTCATTTTTTGTGCACTTTTACCTTCAATTCAAATAATAAAGAGTACACATTTCCAATAACTTT<br>GTCTTCAACTTCTAATCCTCCTCATGGTTTACCATCAACGTTAAGGTGGTTTTTCAATTTATTTCAGTTG<br>TATAGAGGACCATTGGATTTGACAATTATCATCACAGGAGCCACTGATGTGGATGGTATGGCCTGGTT<br>TACTCCAGTAGGCCTTGCTGTCGACACCCCTTGGGTGGAAAAGGAGTCAGCTTTGTCTATTGATTATA<br>AAACTGCCCTTGGAGCTGTTAGATTCAATACAAGAAGAACAGGGAATATTCAGATTAGATTGCCATG<br>GTATTCTTATTTGTATGCCGTGTCTGGAGCGTTGGATGGCTTGGGAGATAAGACAGATTCCACATTTGG<br>ATTGGTTTCTATTCAGATTGCAAATTATAATCATTCTGATGAATATTTGTCCTTTAGTTGTTACTTGTCTG<br>TCACAGAACAATCAGAGTTTTATTTTCCTAGAGCTCCATTGAATTCAAATGCTATGTTGTCCACT	816(bp)

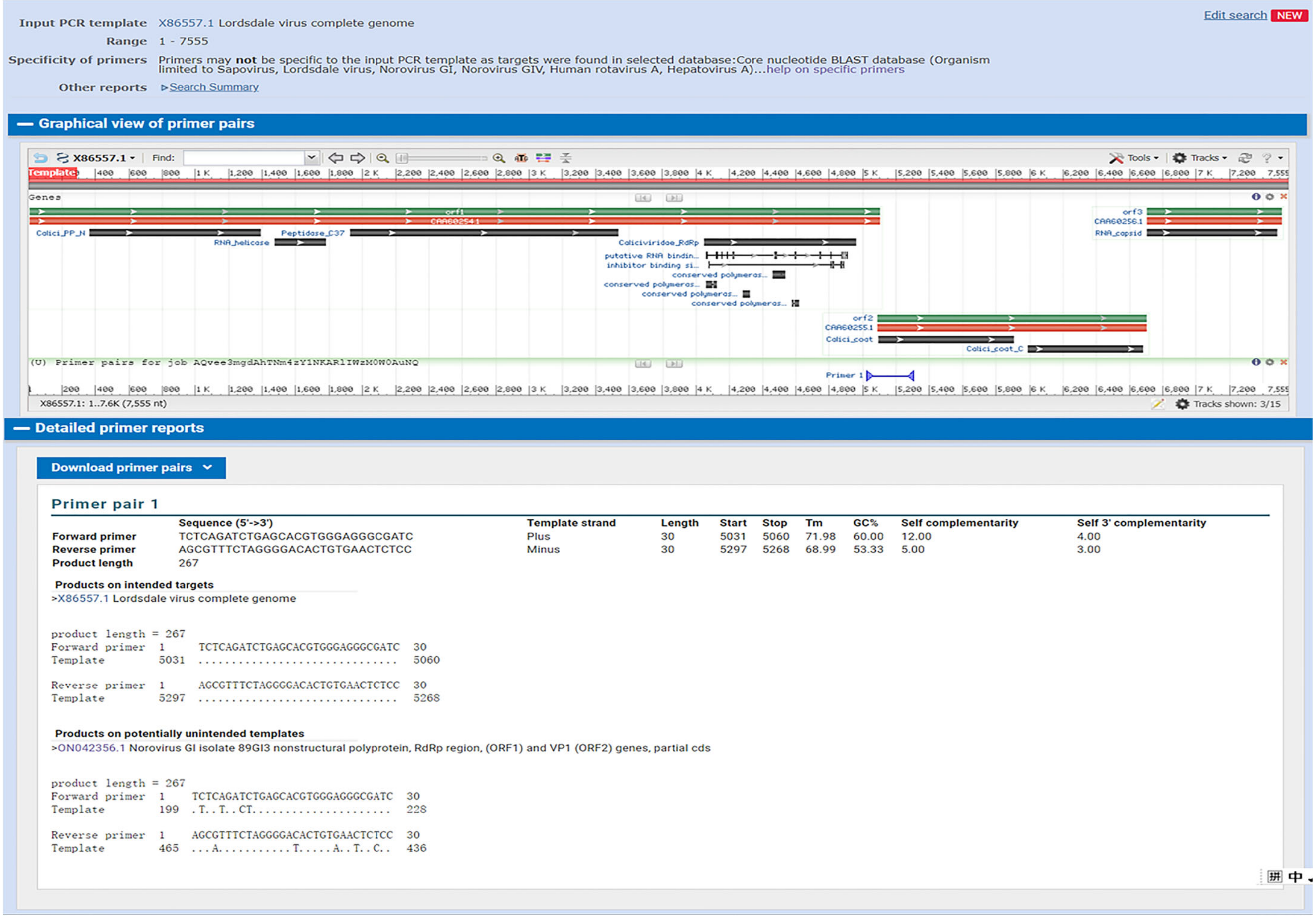

Figure S2. Simulation of the Specificity of the Nf3/NR1 Primer set.

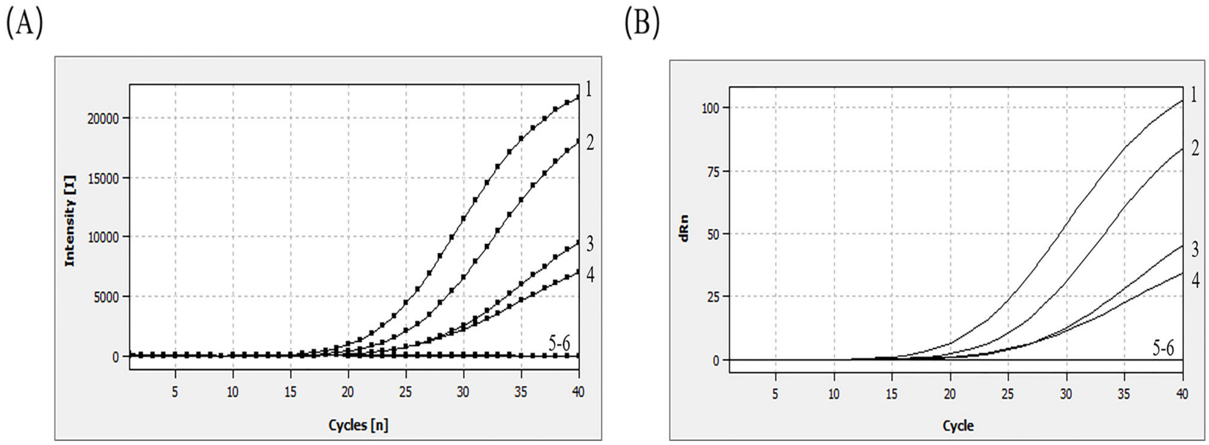

Figure S3. The sensitivity analysis of the MIRA–qPCR at low concentrations (A and B) 1-5: the corresponding concentration of norovirus GII is 1.62 x 10^{3}~10^{-1} copies/μL, 6: negative control; (B) The Ct plot was calculated to correspond to the (A) amplification plot.

(A)

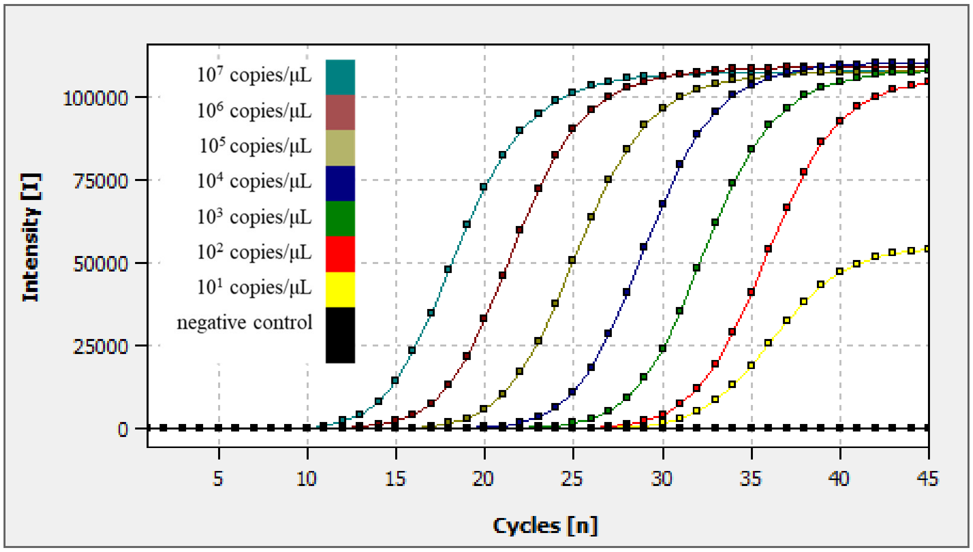
Intensity [I]
100000
75000
50000
25000
0
5
10
15
20
25
30
35
40
45
Cycles [n]
10⁷ copies/μL
10⁶ copies/μL
10⁵ copies/μL
10⁴ copies/μL
10³ copies/μL
10² copies/μL
10¹ copies/μL
negative control

(B)

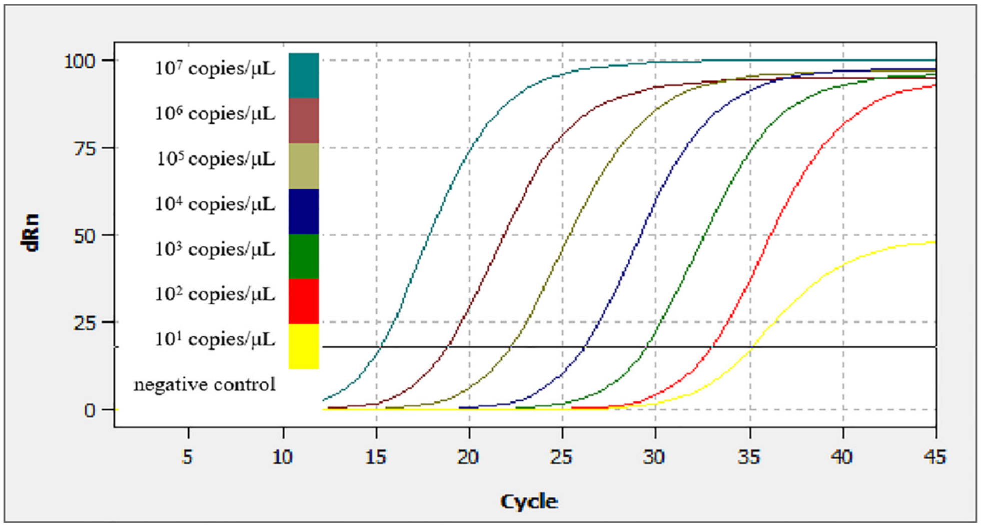
dRn
100
75
50
25
0
5
10
15
20
25
30
35
40
45
Cycle
10⁷ copies/μL
10⁶ copies/μL
10⁵ copies/μL
10⁴ copies/μL
10³ copies/μL
10² copies/μL
10¹ copies/μL
negative control

(C)

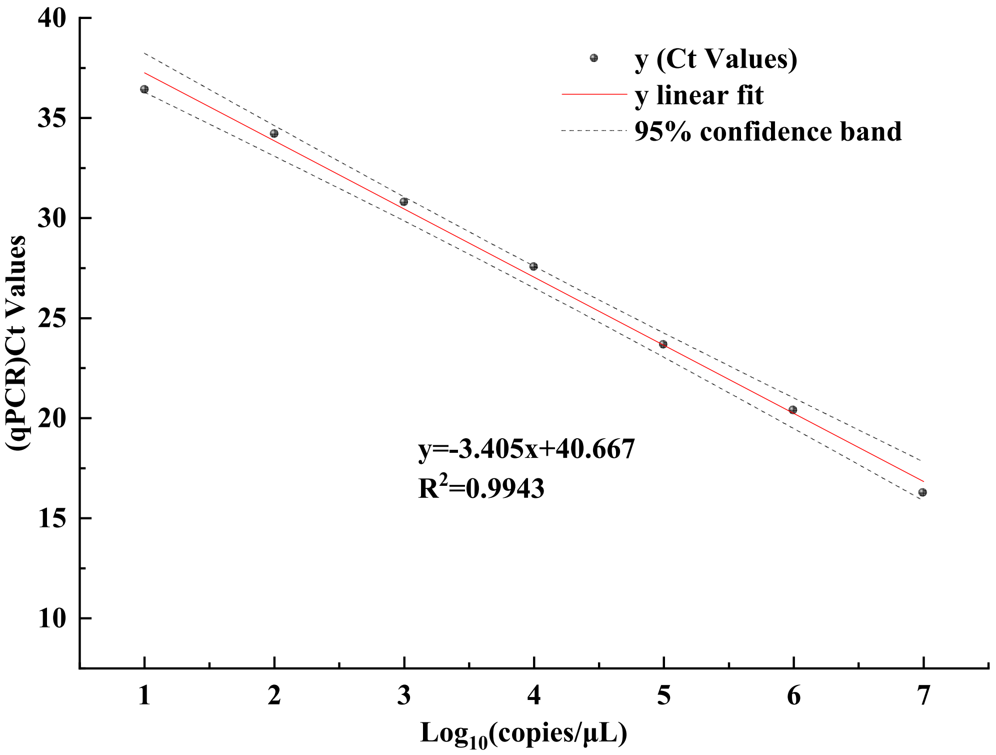

Figure S4. The LOD-relevant results of norovirus GIIusing qPCR (A) The amplification curves showed the LOD of qPCR. (B) The Ct plot was calculated to correspond to the (A) amplification plot. (C) The linear regression standard curve between the Ct values and the logarithm concentrations of norovirus GII as well as the 95% confidence band were obtained using the software Origin 2021.
